# Clinical Performance of a New Soluble CD14-Subtype Immunochromatographic Test for Whole Blood Compared with Chemiluminescent Enzyme Immunoassay: Use of Quantitative Soluble CD14-Subtype Immunochromatographic Tests for the Diagnosis of Sepsis

**DOI:** 10.1371/journal.pone.0143971

**Published:** 2015-12-01

**Authors:** Masayuki Sato, Gaku Takahashi, Shigehiro Shibata, Makoto Onodera, Yasushi Suzuki, Yoshihiro Inoue, Shigeatsu Endo

**Affiliations:** Department of Critical Care Medicine, Iwate Medical University, Uchimaru, Morioka, Iwate, Japan; University of Pittsburgh, UNITED STATES

## Abstract

We previously reported that a soluble CD14-subtype (sCD14-ST) immunochromatographic test (ICT) for plasma is more convenient than chemiluminescent enzyme immunoassay (CLEIA), but plasma separation makes bedside measurements difficult. We developed a new sCD14-ST ICT for whole blood and investigated whether quantitative determinations of sCD14-ST by ICT were useful for diagnosing sepsis and severe sepsis/septic shock. We studied 20 patients who fulfilled two or more systemic inflammatory response syndrome (SIRS) criteria and 32 patients who had been diagnosed with sepsis or severe sepsis/septic shock. Whole blood was collected on day 0 (on admission) and day 7, and the sCD14-ST concentration was quantitatively measured by CLEIA and ICT for whole blood. The patients’ Acute Physiology and Chronic Health Evaluation (APACHE) II, Sequential Organ Failure Assessment (SOFA), and Mortality in Emergency Department Sepsis (MEDS) scores were also calculated. The cut-off values obtained by the quantitative measurements made by ICT were 464.5 pg/mL for sepsis and 762.7 pg/mL for severe sepsis/septic shock (*P* < 0.0001). A Bland–Altman plot showed that no fixed bias or proportional bias was detected between CLEIA and quantitative ICT for whole blood. sCD14-ST concentrations were significantly correlated with APACHE II, SOFA, and MEDS scores (*P* < 0.0001). These results suggest that the new sCD14-ST ICT for whole blood may be a useful tool for the convenient, rapid bedside diagnosis and treatment of sepsis.

## Introduction

Sepsis is a toxic systemic response to infection that progresses to the even more serious conditions of severe sepsis and septic shock accompanied by organ dysfunction [1]. According to the Surviving Sepsis Campaign Guidelines 2012 (SSCG 2012), mortality rates are higher when antimicrobial agents have been administered after septic shock has developed. Targets that should be completed within 3 h and within 6 h have also been clearly identified, and rapid diagnosis and treatment of sepsis has been emphasized [2]. Procalcitonin (PCT), interleukin-6 (IL-6), and tumor necrosis factor-α have previously been used as diagnostic markers for sepsis. PCT, in particular, has been reported to be superior to endotoxin, β-D-glucan, IL-6, and C-reactive protein for differentiating between bacterial infections, including sepsis, and non-bacterial infections [3]. Conversely, PCT is known to be increased in non-infectious systemic inflammatory response syndrome (SIRS) [4–7], and differentiating between non-infectious SIRS and infectious SIRS can be difficult [8].

In recent years, soluble CD14-subtype (sCD14-ST) has been highlighted as a specific marker in infections. In 2005, we reported that quantitatively determined sCD14-ST using a sandwich enzyme-linked immunosorbent assay (ELISA) that showed elevated sCD14-ST levels (specifically increased in sepsis) was a better diagnostic marker than PCT, IL-6, or endotoxin and was strongly correlated with Sequential Organ Failure Assessment (SOFA) scores [9]. However, the assay method is complicated, and because it takes approximately 5–6 h, the time required to make a diagnosis is a disadvantage. In 2011, we quantitatively analyzed sCD14-ST concentrations in only 17 min using the PATHFAST^®^ Presepsin assay system, which is based on a completely automated chemiluminescent enzyme immunoassay (CLEIA) [10].

We previously reported a sCD14-ST immunochromatographic test (ICT) for plasma that allows measurements to be made more conveniently and in 15 min—a shorter time than by CLEIA [11–14]. However, the plasma needs to be separated by centrifugation, and ICT for plasma lacks speed, making bedside measurements difficult. Moreover, the complexity of the assay procedure is a problem. To resolve these issues, we developed a new sCD14-ST ICT for whole blood that can be used to make quantitative determinations using an optical reader.

In this study, we investigated whether quantitative determinations of sCD14-ST by ICT for whole blood would be useful for diagnosing sepsis and severe sepsis/septic shock and whether its quantitative capacity is similar to that provided by CLEIA.

## Material and Methods

The subjects of this study were patients who had been brought to the Critical Care and Emergency Center of Iwate Medical University between October 2013 and April 2014 and who fulfilled two or more SIRS criteria [15], or had been diagnosed with sepsis and severe sepsis/septic shock according to SSCG 2012 [2] and admitted to the intensive care unit or to general hospital wards. Written informed consent was obtained from all patients or their families. This study was approved by the ethics committee of Iwate Medical University and performed according to the Declaration of Helsinki. The exclusion criteria were an age of <18 years and not having obtained informed consent from the patients or their families.

A blood specimen was collected on day 0 (on admission) and day 7, and the sCD14-ST concentration was quantitatively determined by CLEIA and ICT from whole blood. In addition, on day 0 and day 7, the APACHE II score was calculated as an index of the severity of disease [16], the SOFA score was calculated as an index of the severity of organ dysfunction [17], and the Mortality in Emergency Department Sepsis (MEDS) score was calculated as an index of the prognosis of sepsis [18].

### Measurements

Whole blood was collected using collection tubes (Nippon Becton Dickinson, Co., Ltd., Tokyo, Japan) to which ethylenediaminetetraacetate (EDTA)-2K had been added. Within 3 h after collection, the sCD14-ST in 100 μL of whole blood was measured with PATHFAST^®^ Presepsin (LSI Medience Corporation, Tokyo, Japan). At the same time, the sCD14-ST in 120 μL of whole blood was quantitatively determined by ICT.

### Immunochromatographic test

The new sCD14-ST ICT is based on the gold-colloid-based immunochromatography principle, which rapidly detects sCD14-ST in blood specimens. ICT uses a specific gold-colloid-labeled anti-sCD14-ST antibody as a labeled marker for sCD14-ST. Different anti-sCD14-ST antibodies are solid-phased in the test reaction zone and control reaction zone on a cellulose acetate membrane. A whole blood specimen directly added to the sample port is separated into plasma by a filter, and after the plasma has directly formed antigen–antibody complexes with a gold-colloid-labeled anti-sCD14-ST mouse monoclonal antibody, it is transferred as-is to a nitrocellulose membrane, where the sCD14-ST is trapped by an anti-sCD14-ST rabbit polyclonal antibody that has been solid-phased in the test reaction zone. After the excess gold-colloid-labeled anti-sCD14-ST mouse monoclonal antibody is captured by an anti-γ globulin chicken antibody solid-phased in the control reaction zone, it is flushed with excess plasma and absorbed by an absorbent pad. The ICT kit is stored in a refrigerator (2–8°C) and returned to room temperature (20–30°C) prior to use. The kit is rested on a flat surface, and 120 μL of whole blood is added to a sample well by micropipette. After 15 min, sCD14-ST concentrations are automatically and quantitatively determined by DiaScan α (Otsuka Electronics Co., Ltd., Osaka, Japan). The optical system of the reader projects 535-nm light from an LED light source at a 45° angle to the normal direction of the ICT kit. Photosensitive elements arrayed on the normal line detect the reflected light. Measurements are made by placing the ICT kit on a self-propelled table. As the ICT kit is moved horizontally, the absorbance is calculated by continuously measuring the intensity of the reflection of the test reaction zone and converting it to numerical values. The data are then converted into sCD14-ST concentrations and displayed. It takes approximately 12 seconds for a result to be displayed after setting the ICT kit on the table of the reader. DiaScan α is a compact analyzer (width 9.5 cm, height 6.5 cm, depth 16.5 cm, and weight 750 g) driven by dry cells or an alternating current (100–240 V). Therefore, it is portable and can be carried anywhere; for example, to an emergency room or intensive care unit.

### Statistical analysis

Measurements for the control, SIRS, sepsis, and severe sepsis/septic shock groups were tested for normality using the Shapiro–Wilk test. Data are expressed as the mean ± standard deviation (SD) for normally distributed data, and median (25th to 75th percentile) for non-normally distributed data. Comparisons between multiple groups were performed by the Kruskal–Wallis test, Welch’s ANOVA, or Fisher’s exact test. Multiple comparisons were performed by the Steel–Dwass test.

Regression analyses were performed by simple linear regression analysis. Medical outliers were defined as leverage of >0.5. sCD14-ST concentrations measured by CLEIA and ICT for whole blood were compared by the Bland–Altman plot [19]. Cut-off values were calculated from receiver operating characteristic (ROC) curves by means of the Youden index [20]. Correlations were compared by calculating the Spearman rank-order correlation coefficient (ρ). In all tests, a *P* value of <0.05 was considered statistically significant. Statistical analysis was performed using SPSS Statistics 22 software (IBM Corp., Armonk, NY, USA) to analyze the ROC curves; EZR (Saitama Medical Centre, Jichi Medical University, Saitama, Japan; http://www.jichi.ac.jp/saitama-sct/SaitamaHP.files/statmedEN.html), which is a graphical user interface for R (The R Foundation for Statistical Computing, Vienna, Austria, Version 2.2–0) to perform Fisher’s exact test [21]; JMP 11 software (SAS Institute Inc., Cary, NC, USA) to perform the Steel–Dwass test; and Analyse-it Version 2.30 software (Analyse-it Software Ltd., Leeds, UK) to perform other statistical tests.

## Results

### Patient characteristics

**Fig 1:**
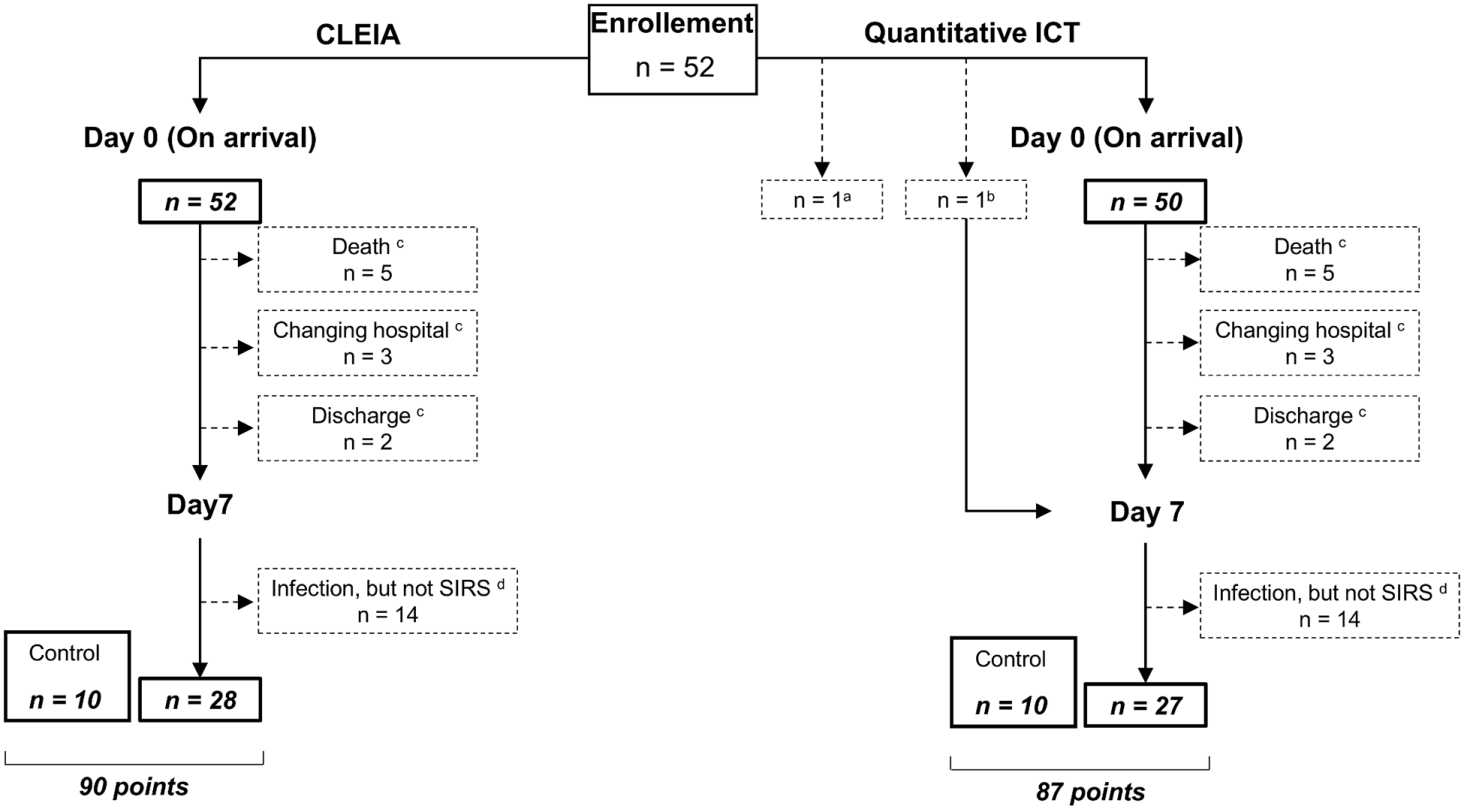
Patient enrollment. Blood was collected from each patient twice: once on day 0 and again on day 7. ^a^Because the reader was not ready in time, quantitative determinations could not be performed by ICT on day 0 and day 7 in one case. ^b^For the same reason, quantitative determinations by ICT could not be performed on day 0 in one case. ^c^By day 7, five patients had died, three had been transferred to another hospital, and two had been discharged. ^d^There were 14 points in time on day 7 when infection was suspected, but the patient did not fulfill at least two criteria for SIRS. The data at those 14 points were excluded from the analysis.

We studied 52 patients (31 males and 21 females) who fulfilled two or more of the diagnostic criteria for SIRS or who were diagnosed with sepsis or severe sepsis/septic shock and were admitted to our department. Twenty patients had been diagnosed with SIRS and 32 patients with sepsis. Of the 32 patients with sepsis, 14 had been diagnosed with sepsis (narrow definition) and 18 with severe sepsis/septic shock. The control group comprised 10 healthy volunteers (5 males and 5 females). Blood was collected from each patient twice, on day 0 and day 7. The data of patients on day 7 who did not have an infection and did not meet two or more diagnostic criteria for SIRS were included in the control group (12 points in CLEIA and 11 points in ICT). As a result, the quantitative determinations by CLEIA were assessed at 90 points (52 points at day 0, 28 points at day 7, and 10 points for controls), and the quantitative determinations by ICT were assessed at 87 points (50 points at day 0, 27 points at day 7, and 10 points for controls) (Fig 1).

The age, sex, APACHE II scores, SOFA scores, MEDS scores, and 28-day mortality rates of the control, SIRS, sepsis, and severe sepsis/septic shock groups are shown in Table 1. No statistically significant differences were found in age or sex among the groups, but statistically significant differences were observed in their APACHE II scores, SOFA scores, MEDS scores, and 28-day mortality rates. Diseases that prompted hospital admission are shown in Table 2.

**Table 1:** Patient characteristics at admission.

	Control (n = 10)	SIRS (n = 20)	Sepsis (n = 14)	Severe sepsis/septic shock (n = 18)	*P* value
Age (years)	61.5 (55.0 to 64.5)	60.9 ± 17.3	74.0 (54.0 to 81.5)	69.8 ± 11.9	0.2225^a^
Male gender [n (%)]	5 (50.0%)	12 (60.0%)	8 (57.1%)	11 (61.1%)	0.9526^b^
SOFA score	No data	2 (1 to 4)	2.1 ± 1.4	7.9 ± 3.7	< 0.0001^a^
APACHE II score	No data	9.3 ± 4.5	11.6 ± 7.1	22.7 ± 7.9	< 0.0001^c^
MEDS score	No data	3 (3 to 6)	5.6 ± 3.4	10.8 ± 4.6	< 0.0001^a^
28-day mortality rate [n (%)]	No data	0 (0.0%)	0 (0.0%)	5 (27.8%)	0.0041^b^

APACHE: Acute Physiology and Chronic Health Evaluation; MEDS: mortality in emergency department sepsis; SIRS: systemic inflammatory response syndrome; SOFA: sequential organ failure assessment.

^a^Kruskal–Wallis test,

^b^Fisher’s exact test,

^c^Welch’s ANOVA.

**Table 2:** Patient disease characteristics at admission.

Disease site	Definitive diagnosis	SIRS	Sepsis	Severe sepsis/septic shock	
					Septic shock
Abdomen	Appendicitis	0	2	0	1
	Cholangitis	0	1	0	2
	Cholecystitis	0	1	2	0
	Intra-abdominal abscess	0	0	1	0
	Liver abscess	0	0	0	1
	Large bowel perforation	0	1	1	1
	Pancreatitis	2	0	0	0
	Perforated gastric ulcer	0	1	0	0
	Perforated duodenal ulcer	0	2	0	0
	Sigmoid volvulus	1	0	0	0
Thorax	Descending necrotizing mediastinitis	0	0	0	1
	Pneumonia	0	4	3	1
Urinary Tract	Pyelonephritis	0	0	1	1
Unknown		0	1	1	0
Other	Burn	6	1	0	0
	Decubitus	0	0	1	0
	Multiple trauma	10	0	0	0
	Systemic lupus erythematosus	1	0	0	0
Total		20	14	10	8

SIRS: systemic inflammatory response syndrome.

### Comparison of sCD14-ST concentrations in different pathological conditions with CLEIA or quantitative ICT

**Fig 2:**
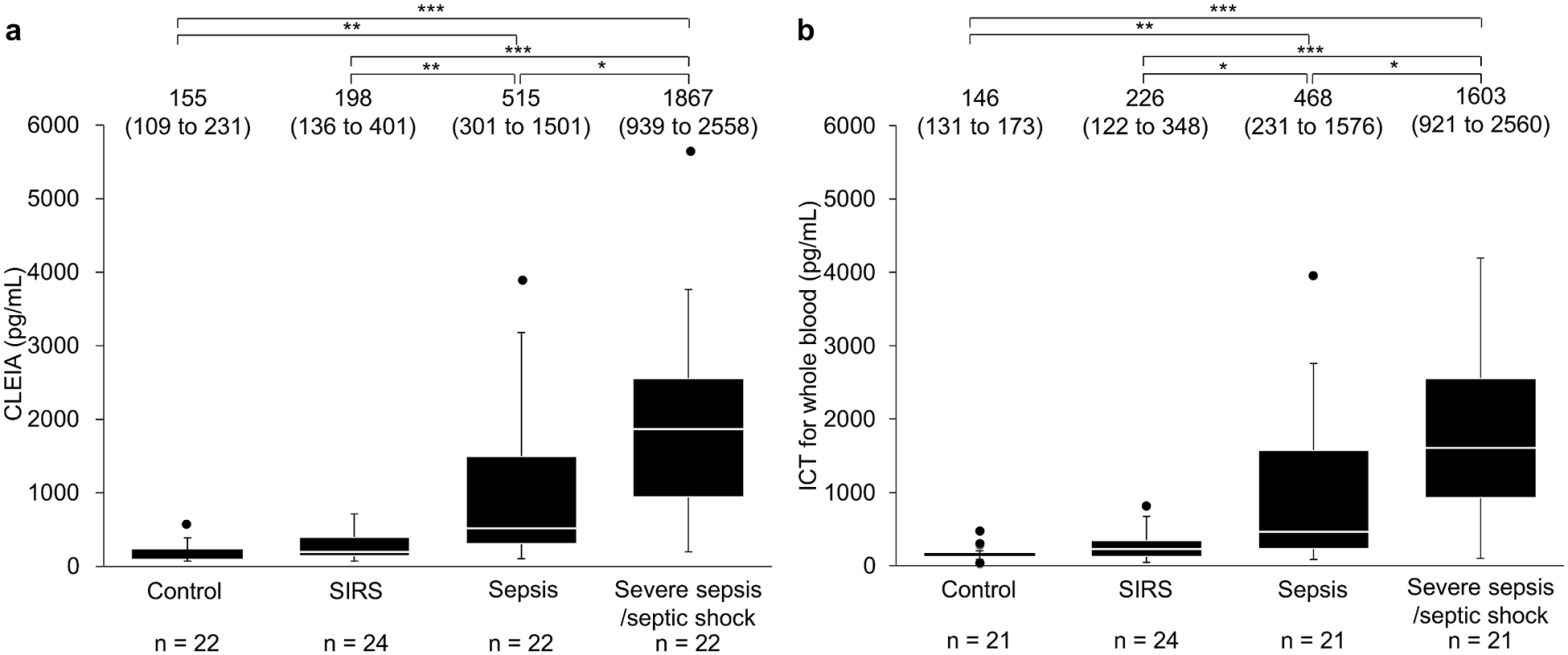
Comparison of sCD14-ST concentration in different pathological conditions with CLEIA or quantitative ICT. (a) CLEIA (n = 90). (b) ICT (n = 87). Box plots show the distribution of sCD14-ST concentrations. White lines represent median, black boxes represent 25th to 75th percentile, whiskers represent range, and filled circles represent outliers. Arabic numerals and those in parenthesis indicate the median and 25th to 75th percentile, respectively. *P < 0.05, **P < 0.01, and ***P < 0.0001 by Steel–Dwass test.

We performed multiple comparisons of the quantitative measurements made by CLEIA and ICT for whole blood in the control, SIRS, sepsis, and severe sepsis/septic shock groups. No statistically significant differences in quantitative measurements by either of the quantitative methods were observed between the control and SIRS groups, but significant differences in the quantitative measurements were observed between the other groups (Fig 2).

### Scatter plot and Bland–Altman plot for comparison between CLEIA and quantitative ICT

**Fig 3:**
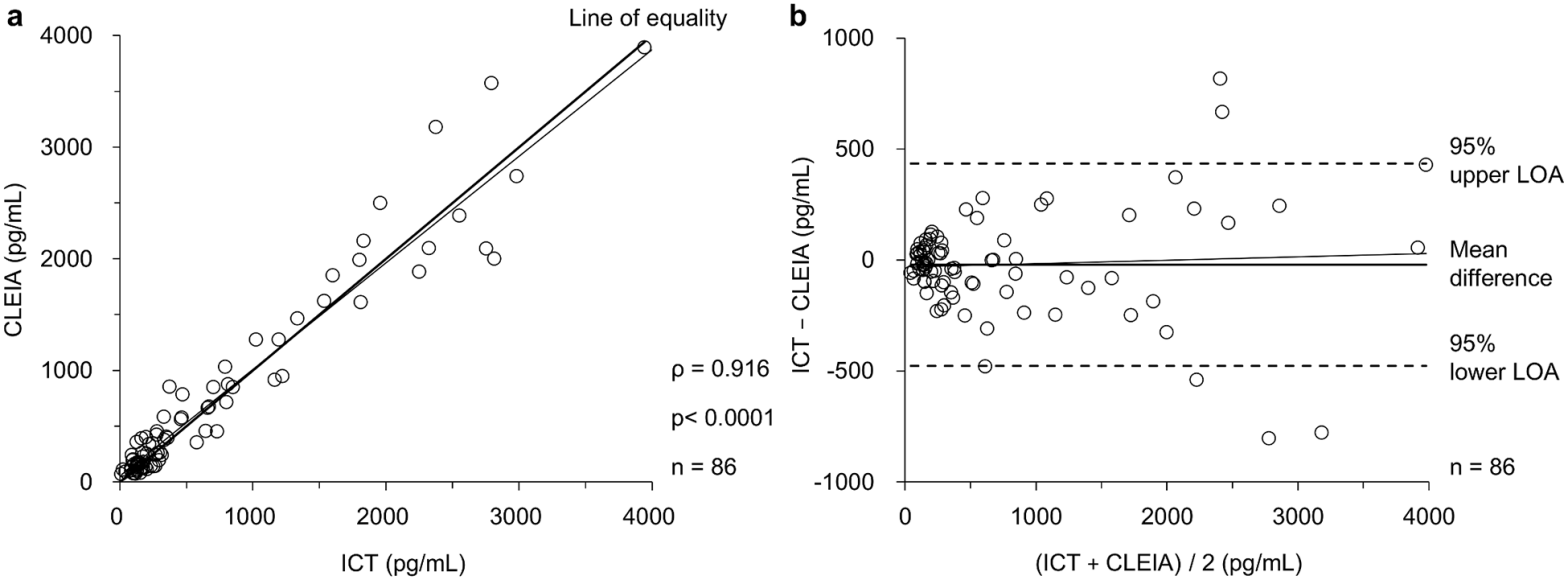
Scatter plot and Bland–Altman plot for comparison between CLEIA and quantitative ICT. (a) Scatter plot between CLEIA and quantitative ICT for whole blood (n = 86). Circle dots represent each point, the thick line represents the line of equality, and the thin line represents the regression line. (b) Bland–Altman plot between CLEIA and quantitative ICT for whole blood (n = 86). Circle dots represent each point, the thick line represents the mean difference, the broken lines represent the 95% upper LOA or the 95% lower LOA, and the thin line represents the regression line.

A scatter plot and Bland–Altman plot [19] were used to assess the equivalence of quantitative measurements made by the two measurement methods (CLEIA and ICT) for whole blood. However, one point was excluded as a medical outlier (CLEIA 5654 pg/mL and ICT 3766 pg/mL). The result of the regression analysis of the quantitative values obtained by CLEIA and ICT was: y = 0.9543x + 54.49 (slope: 95% confidence interval [CI] = 0.9013 to 1.007, *P* < 0.0001; intercept: 95% CI = −8.657 to 117.6, *P* = 0.0899; r^2^ = 0.939), and the correlation coefficient (ρ) was 0.916 (95% CI = 0.873–0.945; *P* < 0.0001) (Fig 3a). Next, the accuracy of the quantitative capacity of CLEIA and ICT was compared by a Bland–Altman plot. The mean difference between the measurements obtained by CLEIA and ICT was −20.50 pg/mL (95% CI = −70.411 to 29.401). The 95% upper limit of agreement (LOA) was 435.72 pg/mL (95% CI = 350.097–521.336), and the 95% lower LOA was −476.73 pg/mL (95% CI = −562.345 to −391.107). The equation for the regression line in the Bland–Altman plot was: y = 0.01531x − 32.04 (slope: 95% CI = −0.03934 to 0.06997, *P* = 0.5789; intercept: 95% CI = −96.61 to 32.82, *P* = 0.3288; r^2^ = 0.004). A significant difference was not observed (Fig 3b). No fixed bias or proportional bias was detected when ICT was used.

### Cut-off values for diagnosing sepsis and severe sepsis/septic shock

The results for diagnosing sepsis showed that the area under curves (AUCs) of the quantitative determinations by CLEIA and ICT for whole blood were 0.899 (95% CI = 0.833–0.965) and 0.869 (95% CI = 0.789–0.948), respectively, and the cut-off values were 569.5 and 464.5 pg/mL, respectively (Table 3). To diagnose severe sepsis/septic shock, the AUCs of the quantitative determinations by CLEIA and ICT were 0.912 (95% CI = 0.847–0.978) and 0.881 (95% CI = 0.787–0.975), respectively, and the cut-off values were 865.0 and 762.7 pg/mL, respectively (Table 4).

**Table 3:** Cut-off values for diagnosing sepsis with CLEIA or quantitative ICT.

	AUC	Cut-off value	Sensitivity	Specificity	Accuracy	PPV	NPV	LR+	LR−	95% CI
CLEIA	0.899	569.5 pg/dL	72.7%	95.7%	84.4%	94.1%	78.6%	16.73	0.29	0.833 to 0.965
ICT	0.869	464.5 pg/dL	71.4%	91.1%	81.6%	88.2%	77.4%	8.04	0.31	0.789 to 0.948

CI: confidence interval; CLEIA: chemiluminescent enzyme immunoassay; ICT: immunochromatographic test; LR: likelihood ratio; NPV: negative predictive value; PPV: positive predictive value.

**Table 4:** Cut-off values for diagnosing severe sepsis/septic shock with CLEIA or quantitative ICT.

	AUC	Cut-off value	Sensitivity	Specificity	Accuracy	PPV	NPV	LR+	LR−	95% CI
CLEIA	0.912	865.0 pg/dL	86.4%	91.2%	90.0%	76.0%	95.4%	9.79	0.15	0.847 to 0.978
ICT	0.881	762.7 pg/dL	85.7%	87.9%	87.4%	69.2%	95.1%	7.07	0.16	0.787 to 0.975

CI: confidence interval; CLEIA: chemiluminescent enzyme immunoassay; ICT: immunochromatographic test; LR: likelihood ratio; NPV: negative predictive value; PPV: positive predictive value.

### Correlations between sCD14-ST concentrations and each index of disease severity with CLEIA or quantitative ICT

Correlations between the quantitative measurements by CLEIA and ICT for whole blood and severity were assessed. sCD14-ST concentrations obtained by CLEIA and ICT were significantly correlated with APACHE II, SOFA, and MEDS scores, and the correlation coefficient (ρ) was 0.5 to 0.6 (*P* < 0.0001) (Table 5).

**Table 5:** Correlations between sCD14-ST concentrations and each index of disease severity with CLEIA or quantitative ICT.

	APACHE II score	SOFA score	MEDS score
CLEIA	0.5939 (*P* < 0.0001)	0.5704 (*P* < 0.0001)	0.6369 (*P* < 0.0001)
ICT	0.5781 (*P* < 0.0001)	0.5579 (*P* < 0.0001)	0.5644 (*P* < 0.0001)

APACHE: Acute Physiology and Chronic Health Evaluation; CLEIA: chemiluminescent enzyme immunoassay; ICT: immunochromatographic test; MEDS: mortality in emergency department sepsis; SOFA: sequential organ failure assessment.

## Discussion

sCD14-ST has been shown to increase specifically in infections and has been proven as a useful marker for the early diagnosis of sepsis [9, 10]. CLEIA allows for measurements in only 17 min, a shorter time than the earlier developed ELISA. Although CLEIA is superior in terms of convenience and speed because it is a fully automated bench-top machine, it may be inferior in terms of portability and economy. We therefore developed an ICT for whole blood that is superior to CLEIA in terms of portability and economy.

Our study suggests that the quantitative determination method by ICT for whole blood is useful for diagnosing both sepsis and severe sepsis/septic shock. Fig 2 shows that no statistically significant differences were observed in the measurements made by either of the quantitative methods between the control and SIRS groups, but significant differences in quantitative measurements were observed between the other groups. This suggests that we can diagnose sepsis and severe sepsis/septic shock and that importantly, we can distinguish between SIRS and sepsis and between SIRS and severe sepsis/septic shock using both CLEIA and ICT.

The AUCs of the ROC curves for the quantitative determinations by CLEIA and ICT were all greater than 0.8 (Tables 3 and 4) and were favorable for diagnosing sepsis. In earlier reports, the optimal cut-off values for diagnosing sepsis by CLEIA ranged from 317 to 647 pg/mL [22–28]. In this study, the optimal cut-off values were 569.5 and 464.5 pg/mL for CLEIA and ICT, respectively (Table 3); these values were consistent with earlier reports but slightly lower than the result obtained by CLEIA. The reason for the low quantitative measurements made by ICT appears to be that the mean difference between the values obtained by CLEIA and ICT was −20.50 pg/mL (95% CI = −70.411 to 29.401) in the Bland–Altman plot.

In this study, sCD14-ST concentrations obtained by ICT for whole blood were correlated with the APACHE II, SOFA, and MEDS scores, and the correlation coefficients were approximately 0.5 to 0.6 (Table 5). As previously reported, we found that the sCD14-ST concentrations are useful for assessing severity and determining prognosis [9, 10, 23, 25].

The ICT kit used in this study can serve as a useful tool that allows the convenient and rapid bedside diagnosis and treatment of sepsis. The ICT kit developed in this study enables quantitative determinations, and its long shelf life and low cost may enable it to be used for the diagnosis and treatment of sepsis in a larger number of institutions.

In conclusion, sCD14-ST ICT may be a useful tool for convenient, rapid bedside diagnosis and treatment of sepsis. These findings should be confirmed in future multicenter studies involving larger numbers of patients.

## Supporting Information

S1 Table Anonymous data set of 52 patients and 10 healthy volunteers (controls). Nos. 1–52 are patients’ data and Nos. 53–62 are healthy volunteers’ data (controls). ^a^Because the reader was not ready in time, quantitative determinations could not be performed by ICT on day 0 and day 7 in one case. ^b^For the same reason, quantitative determinations by ICT could not be performed on day 0 in one case. ^c^By day 7, five patients had died, three had been transferred to another hospital, and two had been discharged. There were 14 points in time on day 7 when infection was suspected, but the patient did not fulfill at least two criteria for SIRS. The data at those 14 points were excluded from the analysis.
(XLSX)
